# Short total sleep duration and poor sleep quality might be associated with asthenozoospermia risk: A case-control study

**DOI:** 10.3389/fphys.2022.959009

**Published:** 2022-10-05

**Authors:** Xiaoying Li, Xiaobin Wang, Qijun Wu, Renhao Guo, Xu Leng, Qiang Du, Bochen Pan, Yuhong Zhao

**Affiliations:** ^1^ Department of Clinical Epidemiology, Shengjing Hospital of China Medical University, Shenyang, China; ^2^ Clinical Research Center, Shengjing Hospital of China Medical University, Shenyang, China; ^3^ Key Laboratory of Precision Medical Research on Major Chronic Disease, Liaoning, China; ^4^ Center for Reproductive Medicine, Shengjing Hospital of China Medical University, Shenyang, China

**Keywords:** association, asthenozoospermia, case-control study, risk, sleep

## Abstract

Sleep has been related to a variety of health outcomes. However, no association between sleep and asthenozoospermia has been reported. The aim of this study is to first investigate the relationship between sleep status and asthenozoospermia risk. A case-control study, including 540 asthenozoospermia cases and 579 controls, was performed from June 2020 to December 2020 in the infertility clinic from Shengjing Hospital of China Medical University. Data on sleep status were collected by Pittsburgh sleep quality index questionnaires and asthenozoospermia was diagnosed based on the World Health Organization guidelines. Odds ratio (OR) with 95% confidence interval (95% CI) was calculated by logistic regression analysis to assess the aforementioned association. Results of this study demonstrated that compared with total sleep duration of 8–9 h/day, < 8 h/day was related to asthenozoospermia risk (OR: 1.44, 95% CI: 1.05–1.99); compared to good sleep quality, poor sleep quality was associated with asthenozoospermia risk (OR: 1.35; 95% CI: 1.04–1.77). There were multiplicative model interaction effects between sleep quality and tea drinking (*p* = 0.04), rotating night shift work (*p* < 0.01) on asthenozoospermia risk. However, we failed to detect any associations between night sleep duration, daytime napping duration, night bedtime, wake-up time, sleep pattern and asthenozoospermia risk. In conclusion, short total sleep duration and poor sleep quality might be related to asthenozoospermia risk. Further well-designed prospective studies are warranted to confirm our findings.

## Introduction

Millions of couples worldwide suffer from infertility (Mascarenhas et al., 2012). Approximately 20%–30% of infertility cases are attributed to male factors, with an additional 20%–30% being ascribed to a combination of male and female factors (Agarwal et al., 2015). Between 1990–2017, the age-standardized male infertility prevalence increased from 710.19 per 100,000 to 768.59 per 100,000, increasing at a rate of 8.224% (Sun et al., 2019). Asthenozoospermia is one of the most common causes of male infertility, accounting for >80% of primary male infertility cases (Curi et al., 2003; Fainberg and Kashanian, 2019; Hwang et al., 2021). Several studies have indicated that asthenozoospermia is due to energy deficiency and structural abnormalities of sperm (du Plessis et al., 2015; Sironen et al., 2020). Numerous reasons may contribute to asthenozoospermia, such as varicocele (Zhu et al., 2018), oxidative stress (Nowicka-Bauer et al., 2018), genetic factors (Li and Chen, 2021), environmental pollutants (Benoff et al., 2008), nutrition (Moghadam et al., 2020), and lifestyle (Gaur et al., 2010).

In recent decades, several studies have investigated the association between sleep and semen quality (Chen et al., 2016; Liu et al., 2017; Chen et al., 2020; Hvidt et al., 2020; Demirkol et al., 2021). For example, Chen et al. (Chen et al., 2020) investigated the association between sleep status and semen quality among healthy men screened as potential sperm donors. The results indicated that long and short sleep duration, as well as poor sleep quality, might impair semen quality parameters. The study of Hvidt et al. (2020) found that normal sleep duration was related to self-reported normal semen quality among men seeking fertility treatment. In this study, semen quality was reported by the participants as “normal” or “reduced” according to the measuring results at the fertility clinics in accordance with the specification of the World Health Organization (WHO) within 3 months prior to inclusion. A prospective study of healthy men in China demonstrated that both excessively long and short sleep duration was related to impaired sperm health (Liu et al., 2017). A cohort study of male college students found an inverse U-shaped effect of sleep duration on semen quality, indicating that both restricted and excessive sleep might have a detrimental influence on semen quality (Chen et al., 2016). A study of men attending the infertility clinic, indicated that infertile shift workers had poor sleep quality and their percentage of normal semen morphology was lower (Demirkol et al., 2021). However, several studies failed to detect an association between sleep and semen quality (Wogatzky et al., 2012; Pokhrel et al., 2019). For example, Wogatzky et al. (2012) found that sleep habits had no effect on sperm among male patients who undergo assisted reproductive technologies. Pokhrel et al. (2019) indicated that sleep duration was not associated with any semen parameters among young Chinese men.

Previous studies have been limited to the effects of sleep on sperm quality parameters. To the best of our knowledge, there is no report on the association between sleep status and asthenozoospermia risk. Sleep is a vital exposure that can affect public health (Yu et al., 2021). Investigating the relationship between sleep status and asthenozoospermia risk may contribute to exploring the complex etiology of asthenozoospermia. To this end, we conducted this hospital-based case-control study to explore whether sleep status were associated with asthenozoospermia risk in Chinese men.

## Materials and methods

### Study design and population

This hospital-based case-control study was performed from June to December 2020; all participants were from the infertility clinic of Shengjing Hospital of China Medical University. We included patients diagnosed with asthenozoospermia, which was defined as the percentage of progressively motile spermatozoa below the lower reference limit (32%) according to the 5th edition of the WHO Laboratory Manual for the Examination and Processing of Human Semen (World Health Organization, 2010). The diagnosis of asthenozoospermia was made when two or more separate semen analysis results had met the criterion. On the other hand, we excluded cases with a varicocele history. We recruited men with normal semen as eligible controls (≥39 ×10^6^ of total sperm count, ≥ 32% progressive motility, ≥ 40% total motility, and ≥4% normal morphology). Finally, 540 asthenozoospermia cases and 579 controls were included in the study. This study was approved by the ethical committee of Shengjing Hospital of China Medical University, and all participants provided written informed consent. This study was conducted in accordance with the Declaration of Helsinki.

### Data collection and sleep quality assessment

Information on sleep status and potential confounding factors (age; physical activity; electronic product use; abstinence time; smoking; alcohol, tea, and coffee drinking; educational level; annual family income; and rotating night shift work) were collected through a standard questionnaire. Data on height and weight were collected by physical examination, and the body mass index (BMI) was calculated as a potential confounding factor. The Pittsburgh sleep quality index (PSQI) score was calculated based on 19 items in the PSQI questionnaire, with good sleep quality being defined as a total PSQI score ≤5.0 (Buysse et al., 1989). Night sleep duration corresponds to actual sleep duration per night. Participants were asked if they took a midday nap and had to reply with a “yes” or a “no.” If the answer was affirmative, we asked for the usual midday nap duration. Total sleep duration corresponds to the sum of daytime napping and night sleep duration. Similar to the study of Chen et al. (2020) (for night sleep duration, 7.5–8 h/day as a reference; for total sleep duration, 8–8.5 h/day as a reference), moderate sleep duration was used as a reference in the present study. In addition, considering the very small number of participants who slept 7.5–8 h per night (5.00%) or 8–8.5 h per day (11.71%), a night sleep duration of 7.5–8.5 h/day and a total sleep duration of 8–9 h/day were taken as the reference in this study. Similar to the study of Hvidt et al. (2020), “before 22:30” was used as a reference of night bedtime in this study. Correspondingly, “before 6:30” was used as a reference for wake-up time. Further, sleep patterns were divided into four groups based on night bedtime and wake-up time: early bed-early rise (before 22:30 and before 6:30, reference group), early bed-late rise (before 22:30 and after 6:30), late bed-early rise (after 22:30 and before 6:30), and late bed-late rise (after 22:30 and after 6:30).

### Definition of confounding factors

Smoking was defined as at least one cigarette per day for more than 6 months; alcohol, tea, or coffee drinking was defined as at least once per week for more than 6 months; educational level was defined as “senior high school/technical secondary school or below” and “junior college/university or above”; annual family income was defined as < 100 and ≥100 RMB thousand yuan; rotating night shift work was defined as ≥ 3 night shifts per month within the last year (Xue et al., 2021). The cut-off values of age and BMI used in stratified analyses were 30 years old and 24 kg/m^2^, respectively; the cut-off values of physical activity and electronic product used in stratified analyses were their respective mean values in controls.

### Semen analyses

This study required all participants to maintain abstinence for 3–7 days. Semen samples were collected by the masturbation method without condoms and lubricants at a dedicated semen collection room. The liquefied period lasted less than 60 min. Semen is classified into four motility grades (i.e. rapidly progressive, slowly progressive, non-progressive or immotile), according to the WHO laboratory manual ("WHO Laboratory Manual for the Examination and Processing of Human Semen, 5th edn. Geneva: World Health Organization. In: World Health Organization, 2010”). We directly measured the ejaculate volume and used the standard pH test strips to measure the semen pH. WLJY9000 (Beijing Weili New Century Science & Tech. Deve. Co. Ltd. Beijing, China), a computer-aided sperm analysis equipment, was used to measure semen concentration as well as the percentage of each motility grade. We used Flow Cytometer BriCyte E6 (Mindray, Shenzhen, China) to evaluate DNA fragmentation in sperms according to the instructions. Briefly, after the fixation of sperm with a low pH buffer, sperm was stained with acridine orange and measured at 488 nm wave length. For each measurement, 5,000 to 10,000 sperms were required. The gating parameters for the measurement included FSC at 500 (electric voltage), SSC at 600, FL1 (Green) at 310, and FL3 (Red) at 410. Sperm smear was stained with the Papanicolaou method and morphology of the sperms was observed under an optical microscope. Normal semen reference values were determined by WHO criteria ("WHO Laboratory Manual for the Examination and Processing of Human Semen, 5th edn. Geneva: World Health Organization. In: World Health Organization, 2010”). External quality control measures were applied throughout this study by joining a national quality control program on semen analysis, which was organized by the Society of Reproductive Medicine, Chinese Medical Association. In this program, we measured the control samples from the Central Lab and sent the target results back for evaluation and monitor. This program can help identify deviations from the norm and ensure the quality of the experiment.

### Statistical analyses

Continuous variables were presented as mean ± standard deviation or median (interquartile range) and analyzed using Student’s t test or non-parametric test based on whether normality was satisfied. Categorical variables were presented as number (percentage) and analyzed using chi-square test. To assess the association between sleep status and asthenozoospermia risk, odds ratio (OR) with 95% confidence intervals (95% CI) was calculated by unconditional logistic regression. A crude logistic regression model without adjustments was applied, and two adjusted models were performed. Model 1 was adjusted for age and body mass index, while model 2 was additionally adjusted for physical activity, electronic product use, abstinence time, smoking, alcohol, tea, and coffee drinking, educational level, annual family income, and rotating night shift work based on model 1. For statistically significant associations, further subgroup analysis and interaction effect analysis were conducted based on potential confounding factors. Multiplicative model interaction effect analysis was conducted using unconditional multiple logistic regression, including the multiplicative interaction term as well as all the covariates in model 2 above. Additive model interaction effects were assessed based on Tomas Andersson’s study and did not exist if the 95%CIs of the relative excess risk due to interaction or the attributable proportion contained 0, or 95%CIs of the synergy index contained 1 (Andersson et al., 2005). Non-linear associations between sleep duration, sleep quality and asthenozoospermia risk were assessed using restricted cubic spline (Gauthier et al., 2020). All statistical analyses were performed using SAS version 9.4 statistical software (SAS Institute Inc. Cary, NC, United States). A two-tailed *p*-value was used and *p* < 0.05 was considered statistically significant.

## Results

Participant characteristics were summarized in Table 1. The distribution of age, abstinence time, and alcohol drinking between cases and controls was statistically different. Compared with controls, the cases were older and more likely to be non-drinkers.

**TABLE 1:** Basic characteristics of participants.

Characteristics	Asthenozoospermia (*n* = 540)	Normal (*n* = 579)	*p* Value
Age (years)	32.50 (30.00, 36.00)	32.00 (29.00, 34.00)	**< 0.05**
Body mass index (kg/m^2^)	26.23 (23.81, 28.73)	25.95 (23.36, 28.73)	0.23
Physical activity (MET/hours/week)	131.33 (100.45, 216.07)	128.15 (97.45, 226.67)	0.88
Electronic product use^a^ (hours/week)	28.00 (18.50, 40.00)	28.00 (20.00, 40.00)	0.64
Abstinence time (days)	4.00 (3.00, 5.00)	4.00 (3.00, 5.00)	**< 0.05**
Smoking (n, %)			0.15
No	280 (51.85)	275 (47.50)	
Yes	260 (48.15)	304 (52.50)	
Alcohol drinking (n, %)			**< 0.05**
No	342 (63.33)	331 (57.17)	
Yes	198 (36.67)	248 (42.83)	
Tea drinking (n, %)			0.33
No	348 (64.44)	389 (67.18)	
Yes	192 (35.56)	190 (32.82)	
Coffee drinking (n, %)			0.94
No	495 (91.67)	530 (91.54)	
Yes	45 (8.33)	49 (8.46)	
Educational level (n, %)			0.25
Senior high school/technical secondary school or below	192 (35.56)	225 (38.86)	
Junior college/university or above	348 (64.44)	354 (61.14)	
Annual family income (RMB thousand yuan), (n, %)			0.87
<100	302 (55.93)	321 (55.44)	
≥100	238 (44.07)	258 (44.56)	
Rotating night shift work (n, %)			0.72
No	473 (87.59)	503 (86.87)	
Yes	67 (12.41)	76 (13.13)	
Night bedtime (hour in 24-h format)	23.00 (22.00, 23.00)	23.00 (22.00, 23.00)	0.86
Wake-up time (hour in 24-h format)	6.83 (6.00, 7.50)	7.00 (6.00, 7.50)	0.45
Night sleep duration (hours/day)	7.00 (6.50, 8.00)	7.00 (6.50, 8.00)	0.84
Taking a nap during the day (n, %)			0.48
No	223 (41.30)	227 (39.21)	
Yes	317 (58.70)	352 (60.79)	
Daytime napping duration^b^ (minutes/day)	40.00 (30.00, 60.00)	40.00 (30.00, 60.00)	0.57
Total sleep duration (hours/day)	7.50 (7.00, 8.33)	8.00 (7.17, 8.50)	0.06
PSQI score	4.00 (3.00, 6.00)	4.00 (3.00, 6.00)	0.13

MET, metabolic equivalent; PSQI, pittsburgh sleep quality index.

a Electronic products include television, computer, mobile phone, iPad and so on.

b Daytime napping duration among participants who take a nap during the day.

The bold values indicated statistical significance.

Associations between sleep status and asthenozoospermia risk were shown in Table 2. Compared with a total sleep duration between 8–9 h/day, a total sleep duration <8 h/day had an association with asthenozoospermia risk (OR: 1.44, 95% CI: 1.05–1.99). Poor sleep quality was related to the risk of asthenozoospermia compared to good sleep quality (OR: 1.35, 95% CI: 1.04–1.77). However, every one-point increase in the PSQI was not significantly associated with asthenozoospermia risk. Additionally, we failed to detect any associations between night sleep duration, daytime napping duration, night bedtime, wake-up time, sleep pattern and asthenozoospermia risk.

**TABLE 2:** Associations between sleep status and asthenozoospermia risk.

Sleep parameters	Cases (n, %)	Controls (n, %)	OR (95%CI)^a^	OR (95%CI)^b^
Total sleep duration (hours/day)				
<8	404 (74.81)	395 (68.22)	**1.44 (1.05, 1.98)**	**1.44 (1.05, 1.99)**
≥8 and <9	82 (15.19)	116 (20.04)	1.00 (Ref)	1.00 (Ref)
≥9	54 (10.00)	68 (11.74)	1.19 (0.75, 1.88)	1.23 (0.77, 1.95)
Night sleep duration (hours/day)				
<7.5	353 (65.37)	380 (65.63)	0.94 (0.72, 1.23)	0.91 (0.69, 1.20)
≥7.5 and <8.5	146 (27.04)	155 (26.77)	1.00 (Ref)	1.00 (Ref)
≥8.5	41 (7.59)	44 (7.60)	1.07 (0.66, 1.74)	1.12 (0.68, 1.83)
Daytime napping duration (hours/day)				
No (0 h/day)	223 (41.30)	227 (39.21)	1.00 (Ref)	1.00 (Ref)
Yes (>0 h/day)	317 (58.70)	352 (60.79)	0.89 (0.70, 1.13)	0.88 (0.69, 1.13)
*p* for the change of every 30 min			0.42	0.43
Night bedtime (hour in 24-h format)				
Before 22:30	242 (44.81)	263 (45.42)	1.00 (Ref)	1.00 (Ref)
After 22:30	298 (55.19)	316 (54.58)	1.01 (0.79, 1.28)	1.03 (0.80, 1.33)
*p* for the change of every 30 min			0.47	0.53
Wake-up time (hour in 24-h format)				
Before 6:30	254 (47.04)	263 (45.42)	1.00 (Ref)	1.00 (Ref)
After 6:30	286 (52.96)	316 (54.58)	0.99 (0.78, 1.25)	1.02 (0.80, 1.31)
*p* for the change of every 30 min			0.64	0.93
Sleep quality				
Good (PSQI score ≤5)	359 (66.48)	424 (73.23)	1.00 (Ref)	1.00 (Ref)
Poor (PSQI score >5)	181 (33.52)	155 (26.77)	**1.33 (1.03, 1.72)**	**1.35 (1.04, 1.77)**
*p* for the change of every 1 score			0.27	0.22
Sleep pattern				
Early bed-early rise	162 (30.00)	166 (28.67)	1.00 (Ref)	1.00 (Ref)
Early bed-late rise	80 (14.81)	97 (16.75)	0.91 (0.63, 1.32)	0.93 (0.64, 1.35)
Late bed-early rise	92 (17.04)	97 (16.75)	0.95 (0.66, 1.36)	0.94 (0.65, 1.36)
Late bed-late rise	206 (38.15)	219 (37.83)	0.99 (0.74, 1.32)	1.03 (0.76, 1.42)

CI, confidence interval; OR, odds ratio; PSQI, pittsburgh sleep quality index.

a Model 1: Adjusted for age and body mass index.

b Model 2: Adjusted for age, body mass index, physical activity, electronic product use, abstinence time, smoking, alcohol drinking, tea drinking, coffee drinking, educational level, annual family income, and rotating night shift work.

The bold values indicated statistical significance.

Subgroup analysis stratified by relevant characteristics showed that the direction of the majority of the findings was consistent with the main results (Table 3; Supplementry Table S1). Importantly, compared with good sleep quality, we observed slightly stronger associations between poor sleep quality and asthenozoospermia risk in participants with rotating night shift work (OR: 3.52; 95% CI: 1.66–7.77) (Table 3). There were multiplicative model interaction effects between sleep quality and tea drinking (*p* = 0.04), rotating night shift work (*p* < 0.01) on asthenozoospermia risk (Table 3). However, we failed to detect multiplicative model interaction effects between total sleep duration and potential confounding factors, and additive model interaction effects between total sleep duration, sleep quality and potential confounding factors (Supplementary Table S1, S2).

**TABLE 3:** Subgroup analyses of the associations between sleep quality and asthenozoospermia risk.

Variables		Cases (n, %)	Controls (n, %)	OR (95% CI)^a^	*p*_multiplicative interaction_
Age (years)	**< 30**				0.40
	Good (PSQI score ≤5)	83 (72.17)	112 (74.17)	1.00 (Ref)	
	Poor (PSQI score >5)	32 (27.83)	39 (25.83)	1.15 (0.64, 2.05)	
	*p* for the change of every 1 score			0.94	
	**≥ 30**				
	Good (PSQI score ≤5)	276 (64.94)	312 (72.90)	1.00 (Ref)	
	Poor (PSQI score >5)	149 (35.06)	116 (27.10)	**1.46 (1.08, 1.97)**	
	*p* for the change of every 1 score			0.07	
Body mass index (kg/m^2^)	**< 24**				0.78
	Good (PSQI score ≤5)	96 (67.13)	130 (73.45)	1.00 (Ref)	
	Poor (PSQI score >5)	47 (32.87)	47 (26.55)	1.35 (0.82, 2.25)	
	*p* for the change of every 1 score			0.40	
	**≥ 24**				
	Good (PSQI score ≤5)	263 (66.25)	294 (73.13)	1.00 (Ref)	
	Poor (PSQI score >5)	134 (33.75)	108 (26.87)	**1.40 (1.02, 1.91)**	
	*p* for the change of every 1 score			0.30	
Physical activity (MET/hours/week)	**< mean value**				0.48
	Good (PSQI score ≤5)	237 (68.30)	272 (73.91)	1.00 (Ref)	
	Poor (PSQI score >5)	110 (31.70)	96 (26.09)	1.26 (0.90, 1.75)	
	*p* for the change of every 1 score			0.45	
	**≥ mean value**				
	Good (PSQI score ≤5)	122 (63.21)	152 (72.04)	1.00 (Ref)	
	Poor (PSQI score >5)	71 (36.79)	59 (27.96)	**1.61 (1.04, 2.52)**	
	*p* for the change of every 1 score			0.24	
Electronic product use (hours/week)	**< mean value**				0.09
	Good (PSQI score ≤5)	227 (69.63)	249 (73.02)	1.00 (Ref)	
	Poor (PSQI score >5)	99 (30.37)	92 (26.98)	1.13 (0.80, 1.59)	
	*p* for the change of every 1 score			0.80	
	**≥ mean value**				
	Good (PSQI score ≤5)	132 (61.68)	175 (73.53)	1.00 (Ref)	
	Poor (PSQI score >5)	82 (38.32)	63 (26.47)	**1.79 (1.18, 2.74)**	
	*p* for the change of every 1 score			0.09	
Smoking	**No**				0.80
	Good (PSQI score ≤5)	194 (69.29)	210 (76.36)	1.00 (Ref)	
	Poor (PSQI score >5)	86 (30.71)	65 (23.64)	1.44 (0.98, 2.13)	
	*p* for the change of every 1 score			0.61	
	**Yes**				
	Good (PSQI score ≤5)	165 (63.46)	214 (70.39)	1.00 (Ref)	
	Poor (PSQI score >5)	95 (36.54)	90 (29.61)	1.31 (0.91, 1.89)	
	*p* for the change of every 1 score			0.17	
Alcohol drinking	**No**				0.98
	Good (PSQI score ≤5)	234 (68.42)	250 (75.53)	1.00 (Ref)	
	Poor (PSQI score >5)	108 (31.58)	81 (24.47)	1.29 (0.91, 1.84)	
	*p* for the change of every 1 score			0.68	
	**Yes**				
	Good (PSQI score ≤5)	125 (63.13)	174 (70.16)	1.00 (Ref)	
	Poor (PSQI score >5)	73 (36.87)	74 (29.84)	1.38 (0.92, 2.08)	
	*p* for the change of every 1 score			0.20	
Tea drinking	**No**				**0.04**
	Good (PSQI score ≤5)	251 (72.13)	288 (74.04)	1.00 (Ref)	
	Poor (PSQI score >5)	97 (27.87)	101 (25.96)	1.08 (0.77, 1.52)	
	*p* for the change of every 1 score			0.77	
	**Yes**				
	Good (PSQI score ≤5)	108 (56.25)	136 (71.58)	1.00 (Ref)	
	Poor (PSQI score >5)	84 (43.75)	54 (28.42)	**1.86 (1.20, 2.90)**	
	*p* for the change of every 1 score			**0.03**	
Coffee drinking	**No**				0.59
	Good (PSQI score ≤5)	332 (67.07)	389 (73.40)	1.00 (Ref)	
	Poor (PSQI score >5)	163 (32.93)	141 (26.60)	**1.33 (1.01, 1.76)**	
	*p* for the change of every 1 score			0.28	
	**Yes**				
	Good (PSQI score ≤5)	27 (60.00)	35 (71.43)	1.00 (Ref)	
	Poor (PSQI score >5)	18 (40.00)	14 (28.57)	1.43 (0.55, 3.77)	
	*p* for the change of every 1 score			0.74	
Educational level	**Senior high school/technical secondary school or below**				0.12
	Good (PSQI score ≤5)	133 (69.27)	159 (70.67)	1.00 (Ref)	
	Poor (PSQI score >5)	59 (30.73)	66 (29.33)	1.07 (0.69, 1.65)	
	*p* for the change of every 1 score			0.46	
	**Junior college/university or above**				
	Good (PSQI score ≤5)	226 (64.94)	265 (74.86)	1.00 (Ref)	
	Poor (PSQI score >5)	122 (35.06)	89 (25.14)	**1.58 (1.13, 2.21)**	
	*p* for the change of every 1 score			0.34	
Annual family income (RMB thousand yuan)	**< 100**				0.32
	Good (PSQI score ≤5)	205 (67.88)	232 (72.27)	1.00 (Ref)	
	Poor (PSQI score >5)	97 (32.12)	89 (27.73)	1.20 (0.84, 1.72)	
	*p* for the change of every 1 score			0.30	
	**≥ 100**				
	Good (PSQI score ≤5)	154 (64.71)	192 (74.42)	1.00 (Ref)	
	Poor (PSQI score >5)	84 (35.29)	66 (25.58)	**1.55 (1.03, 2.32)**	
	*p* for the change of every 1 score			0.45	
Rotating night shift work	**No**				**<0.01**
	Good (PSQI score ≤5)	329 (69.56)	370 (73.56)	1.00 (Ref)	
	Poor (PSQI score >5)	144 (30.44)	133 (26.44)	1.16 (0.87, 1.55)	
	*p* for the change of every 1 score			0.64	
	**Yes**				
	Good (PSQI score ≤5)	30 (44.78)	54 (71.05)	1.00 (Ref)	
	Poor (PSQI score >5)	37 (55.22)	22 (28.95)	**3.52 (1.66, 7.77)**	
	*p* for the change of every 1 score			0.07	

CI, confidence interval; MET, metabolic equivalent; OR, odds ratio; PSQI, pittsburgh sleep quality index.

a Adjusted ^a^or age, body mass index, physical activity, electronic product use, abstinence time, smoking, alcohol drinking, tea drinking, coffee drinking, educational level, annual family income, and rotating night shift work, unless a certain covariable is the basis of the stratification.

The bold values indicated statistical significance.

We further assessed non-linear associations between sleep duration, sleep quality and asthenozoospermia risk, but found no non-linear association (*p*_non-linear_ > 0.05).

## Discussion

This hospital-based case-control study, including 540 cases and 579 controls, found that total sleep duration and sleep quality were associated with asthenozoospermia risk. Compared to total sleep duration between 8–9 h/day, total sleep duration <8 h/day was associated with asthenozoospermia risk. Compared to good sleep quality, poor sleep quality was related to the risk of asthenozoospermia.

Despite the lack of studies on the association between sleep status and risk of asthenozoospermia, several studies have provided evidence of the association between sleep status and sperm quality (Liu et al., 2017; Chen et al., 2020; Du et al., 2020; Hvidt et al., 2020; Lateef and Akintubosun, 2020). Interestingly, the findings of these previous studies have been controversial. Therefore, our results are consistent only with some of them (Chen et al., 2020; Du et al., 2020; Lateef and Akintubosun, 2020). For example, we found that short total sleep duration (<8 h/day) was related to the risk of asthenozoospermia, compared with a total sleep duration between 8–9 h/day, which corroborated with the findings on sperm quality of Chen et al. (2020). However, Chen et al. (2020) found that long total sleep duration can impair sperm quality, which contradicted the results of our study. This inconsistency might be partly attributed to the limited number of participants (10.90%) with long total sleep duration (≥9 h/day) in our study. In contrast, participants with long total sleep duration (≥9 h/day) accounted for 22.81% in the study by Chen et al. (2020). Our study found that long night sleep duration was not associated with asthenozoospermia risk, consistent with previous findings on sperm quality (Chen et al., 2020; Hvidt et al., 2020). However, the same was true for short night sleep duration in our study, which contradicted the previous findings on sperm quality (Chen et al., 2020; Hvidt et al., 2020). This inconsistency might be because short night sleep duration can be related to certain sperm quality parameters, but not enough to be related to asthenozoospermia risk. In addition, our study found that daytime napping duration was not related to the risk of asthenozoospermia, consistent with the findings on sperm quality of Chen et al. (2020).

Several previous studies indicated sleep quality can be associated with sperm quality, in accord with our results on the association between sleep quality and asthenozoospermia risk (Chen et al., 2020; Du et al., 2020; Lateef and Akintubosun, 2020). However, Hvidt et al. found no relationship between sleep quality and sperm quality. This contradiction may have arisen because of the different methods used for evaluation of semen quality. The evaluation of semen quality was based on the direct laboratory examination in our study, but self-reported data (questionnaire) in Hvidt et al.(2020) study.

Our study detected interaction effects between sleep quality and tea drinking, rotating night shift work on asthenozoospermia risk. Poor sleep quality was related to the risk of asthenozoospermia but only among participants who drank tea and those with rotating night shift work. The exact reason is unclear, but underlying mechanisms might be that tea polyphenols (Qi et al., 2017) and rotating night shift work (Demirkol et al., 2021) have an influence on circadian rhythms that can affect male reproduction (Peterlin et al., 2019).

Potential mechanisms underlying the association between sleep and male infertility might involve many aspects, such as endocrinology, metabolism, and stress. At first, poor sleep may impair secretory activity of the pituitary-testis axis and decrease circulating levels of male sex hormones (Fusco et al., 2021). Then, sleep duration may affect the risk of metabolic syndrome, which is related to lower male reproductive function (Zhou et al., 2020; Che et al., 2021). In addition, abnormal sleep can reduce melatonin production, which has a variety of functions, such as antioxidant stress, anti-aging, anti-inflammatory, and immune regulation (Ferlazzo et al., 2020). Abnormalities in these functions might affect male infertility (Condorelli et al., 2017; Smits et al., 2019).

Our study has some strengths. First, it had a relatively large sample size of both cases (*n* = 540) and controls (*n* = 579), which provided more reliable and precise risk estimates. Notably, we performed numerous subgroup analyses to investigate the aforementioned topic. Second, this study used the standardized PSQI questionnaire reflecting both sleep duration and quality. However, this study also has a few limitations. First, the reproducibility of the PSQI score over time among our study participants failed to be checked. However, some studies indicated that PSQI was reliable over 2–16 weeks (Fabbri et al., 2021). Second, selection and recall biases might exist due to a case-control study design. Information on sleep status and potential confounding factors obtained from questionnaires might be subject to recall bias. Third, this study failed to completely exclude confounding bias because some covariates were unmeasured, such as genetic factors (Ni et al., 1997; Ley et al., 2018), air pollutants (Kumar et al., 2021; Sun et al., 2020), and water and soil pollution (Di Nisio and Foresta, 2019), which might influence the aforementioned associations. Further research is needed to rule out the possibility of residual confounders and better elucidate the association between sleep status and asthenozoospermia risk.

## Conclusion

This hospital-based case-control study demonstrated that short total sleep duration and poor sleep quality might be related to the risk of asthenozoospermia. Further studies with a prospective study design are warranted to confirm our findings.

## Data Availability

The original contributions presented in the study are included in the article/Supplementary Material, further inquiries can be directed to the corresponding authors.
