# Supplementary material for: Short total sleep duration and poor sleep quality might be associated with asthenozoospermia risk: A case-control study
Source: Front Physiol. 2022 Oct 5;13:959009. doi: 10.3389/fphys.2022.959009 (PMC9581216; doi:10.3389/fphys.2022.959009)
Supplement: Supplementary file 1 [file DataSheet1.docx]

Table S1. Subgroup analyses of the associations between total sleep duration and asthenozoospermia risk

| **Variables** | | **Cases (n, %)** | **Controls (n, %)** | **OR (95% CI) ^a^** | ***P* _multiplicative interaction_** |
| --- | --- | --- | --- | --- | --- |
| **Age (years)** | **< 30** |  |  |  | 0.78 |
|  | < 8 | 83 (72.18) | 100 (66.23) | 1.25 (0.63, 2.52) |  |
|  | ≥ 8 and < 9 | 18 (15.65) | 28 (18.54) | 1.00 (Ref) |  |
|  | ≥ 9 | 14 (12.17) | 23 (15.23) | 0.99 (0.38, 2.53) |  |
|  | **≥ 30** |  |  |  |  |
|  | < 8 | 321 (75.53) | 295 (68.93) | **1.46 (1.02, 2.11)** |  |
|  | ≥ 8 and < 9 | 64 (15.06) | 88 (20.56) | 1.00 (Ref) |  |
|  | ≥ 9 | 40 (9.41) | 45 (10.51) | 1.21 (0.70, 2.07) |  |
| **Body mass index (kg/m^2^)** | **< 24** |  |  |  | 0.89 |
|  | < 8 | 113 (79.02) | 118 (66.67) | **1.97 (1.04, 3.90)** |  |
|  | ≥ 8 and < 9 | 16 (11.19) | 34 (19.21) | 1.00 (Ref) |  |
|  | ≥ 9 | 14 (9.79) | 25 (14.12) | 1.38 (0.55, 3.43) |  |
|  | **≥ 24** |  |  |  |  |
|  | < 8 | 291 (73.30) | 277 (68.90) | 1.32 (0.91, 1.92) |  |
|  | ≥ 8 and < 9 | 66 (16.62) | 82 (20.40) | 1.00 (Ref) |  |
|  | ≥ 9 | 40 (10.08) | 43 (10.70) | 1.30 (0.75, 2.26) |  |
| **Physical activity (MET/hours/week)** | **< mean value** |  |  |  | 0.22 |
|  | < 8 | 259 (74.64) | 246 (66.85) | **1.65 (1.11, 2.46)** |  |
|  | ≥ 8 and < 9 | 52 (14.99) | 82 (22.28) | 1.00 (Ref) |  |
|  | ≥ 9 | 36 (10.37) | 40 (10.87) | 1.46 (0.82, 2.60) |  |
|  | **≥ mean value** |  |  |  |  |
|  | < 8 | 145 (75.13) | 149 (70.62) | 1.16 (0.66, 2.05) |  |
|  | ≥ 8 and < 9 | 30 (15.54) | 34 (16.11) | 1.00 (Ref) |  |
|  | ≥ 9 | 18 (9.33) | 28 (13.27) | 0.87 (0.38, 1.94) |  |
| **Electronic product use**  **(hours/week)** | **< mean value** |  |  |  | 0.24 |
|  | < 8 | 246 (75.46) | 238 (69.79) | 1.23 (0.81, 1.87) |  |
|  | ≥ 8 and < 9 | 51 (15.64) | 64 (18.77) | 1.00 (Ref) |  |
|  | ≥ 9 | 29 (8.90) | 39 (11.44) | 0.95 (0.51, 1.76) |  |
|  | **≥ mean value** |  |  |  |  |
|  | < 8 | 158 (73.83) | 157 (65.97) | **1.87 (1.12, 3.16)** |  |
|  | ≥ 8 and < 9 | 31 (14.49) | 52 (21.85) | 1.00 (Ref) |  |
|  | ≥ 9 | 25 (11.68) | 29 (12.18) | 1.61 (0.77, 3.35) |  |
| **Smoking** | **No** |  |  |  | 0.59 |
|  | < 8 | 219 (78.22) | 196 (71.27) | 1.57 (0.98, 2.52) |  |
|  | ≥ 8 and < 9 | 38 (13.57) | 53 (19.27) | 1.00 (Ref) |  |
|  | ≥9 | 23 (8.21) | 26 (9.46) | 1.33 (0.65, 2.75) |  |
|  | **Yes** |  |  |  |  |
|  | < 8 | 185 (71.16) | 199 (65.46) | 1.34 (0.86, 2.10) |  |
|  | ≥ 8 and < 9 | 44 (16.92) | 63 (20.72) | 1.00 (Ref) |  |
|  | ≥ 9 | 31 (11.92) | 42 (13.82) | 1.09 (0.59, 2.02) |  |
| **Alcohol drinking** | **No** |  |  |  | 0.23 |
|  | < 8 | 256 (74.85) | 231 (69.79) | 1.45 (0.95, 2.24) |  |
|  | ≥ 8 and < 9 | 49 (14.33) | 67 (20.24) | 1.00 (Ref) |  |
|  | ≥ 9 | 37 (10.82) | 33 (9.97) | 1.66 (0.90, 3.08) |  |
|  | **Yes** |  |  |  |  |
|  | < 8 | 148 (74.75) | 164 (66.13) | 1.41 (0.85, 2.37) |  |
|  | ≥ 8 and < 9 | 33 (16.67) | 49 (19.76) | 1.00 (Ref) |  |
|  | ≥ 9 | 17 (8.58) | 35 (14.11) | 0.79 (0.37, 1.67) |  |
| **Tea drinking** | **No** |  |  |  | 0.36 |
|  | < 8 | 256 (73.56) | 267 (68.64) | 1.27 (0.86, 1.90) |  |
|  | ≥ 8 and < 9 | 56 (16.09) | 74 (19.02) | 1.00 (Ref) |  |
|  | ≥ 9 | 36 (10.35) | 48 (12.34) | 1.06 (0.60, 1.87) |  |
|  | **Yes** |  |  |  |  |
|  | < 8 | 148 (77.08) | 128 (67.37) | **1.84 (1.04, 3.29)** |  |
|  | ≥ 8 and < 9 | 26 (13.54) | 42 (22.10) | 1.00 (Ref) |  |
|  | ≥ 9 | 18 (9.38) | 20 (10.53) | 1.61 (0.69, 3.79) |  |
| **Coffee drinking** | **No** |  |  |  | 0.80 |
|  | < 8 | 367 (74.14) | 363 (68.49) | 1.38 (0.99, 1.94) |  |
|  | ≥ 8 and < 9 | 77 (15.56) | 105 (19.81) | 1.00 (Ref) |  |
|  | ≥ 9 | 51 (10.30) | 62 (11.70) | 1.23 (0.76, 2.00) |  |
|  | **Yes** |  |  |  |  |
|  | < 8 | 37 (82.22) | 32 (65.31) | 3.58 (0.99, 15.34) |  |
|  | ≥ 8 and < 9 | 5 (11.11) | 11 (22.45) | 1.00 (Ref) |  |
|  | ≥ 9 | 3 (6.67) | 6 (12.24) | 1.36 (0.16, 11.07) |  |
| **Educational level** | **Senior high school/technical secondary school or below** | | | | 0.81 |
|  | < 8 | 141 (73.44) | 153 (68.00) | 1.43 (0.83, 2.48) |  |
|  | ≥ 8 and < 9 | 29 (15.10) | 44 (19.56) | 1.00 (Ref) |  |
|  | ≥ 9 | 22 (11.46) | 28 (12.44) | 1.36 (0.64, 2.91) |  |
|  | **Junior college/university or above** | | |  |  |
|  | < 8 | 263 (75.57) | 242 (68.36) | 1.48 (0.99, 2.23) |  |
|  | ≥ 8 and < 9 | 53 (15.23) | 72 (20.34) | 1.00 (Ref) |  |
|  | ≥ 9 | 32 (9.20) | 40 (11.30) | 1.16 (0.64, 2.12) |  |
| **Annual family income (RMB thousand yuan)** | **< 100** |  |  |  | 0.74 |
|  | < 8 | 209 (69.21) | 212 (66.04) | 1.18 (0.79, 1.79) |  |
|  | ≥ 8 and < 9 | 56 (18.54) | 68 (21.19) | 1.00 (Ref) |  |
|  | ≥ 9 | 37 (12.25) | 41 (12.77) | 1.21 (0.68, 2.16) |  |
|  | **≥ 100** |  |  |  |  |
|  | < 8 | 195 (81.93) | 183 (70.93) | **1.94 (1.15, 3.34)** |  |
|  | ≥ 8 and < 9 | 26 (10.93) | 48 (18.60) | 1.00 (Ref) |  |
|  | ≥ 9 | 17 (7.14) | 27 (10.47) | 1.24 (0.56, 2.73) |  |
| **Rotating night shift work** | **No** |  |  |  | 1.00 |
|  | < 8 | 360 (76.11) | 357 (70.97) | 1.37 (0.97, 1.94) |  |
|  | ≥ 8 and < 9 | 71 (15.01) | 96 (19.09) | 1.00 (Ref) |  |
|  | ≥ 9 | 42 (8.88) | 50 (9.94) | 1.27 (0.75, 2.13) |  |
|  | **Yes** |  |  |  |  |
|  | < 8 | 44 (65.67) | 38 (50.00) | 2.10 (0.86, 5.34) |  |
|  | ≥ 8 and < 9 | 11 (16.42) | 20 (26.32) | 1.00 (Ref) |  |
|  | ≥ 9 | 12 (17.91) | 18 (23.68) | 1.38 (0.45, 4.27) |  |

CI, confidence interval; MET, metabolic equivalent; OR, odds ratio.

^a^ Adjusted for age, body mass index, physical activity, electronic product use, abstinence time, smoking, alcohol drinking, tea drinking, coffee drinking, educational level, annual family income, and rotating night shift work, unless a certain covariable is the basis of the stratification.

Table S2. Additive model interaction effects between total sleep duration, sleep quality and confounding factors on asthenozoospermia risk

| **Variables** | | **RERI (95%CI) ^a^** | **AP (95%CI) ^a^** | **S (95%CI) ^a^** |
| --- | --- | --- | --- | --- |
| **Age (years)** | Total sleep duration | 0.19 (-0.57, 0.95) | 0.11 (-0.38, 0.60) | 1.37 (0.22, 8.70) |
|  | Sleep quality | 0.43 (-0.30, 1.15) | 0.25 (-0.17, 0.66) | 2.46 (0.19, 31.77) |
| **Body mass index (kg/m^2^)** | Total sleep duration | -0.41 (-1.55, 0.74) | -0.20 (-0.67, 0.28) | 0.73 (0.40, 1.34) |
|  | Sleep quality | 0.16 (-0.56, 0.89) | 0.10 (-0.35, 0.56) | 1.39 (0.25, 7.94) |
| **Physical activity (MET/hours/week)** | Total sleep duration | -0.48 (-1.48, 0.52) | -0.30 (-0.91, 0.31) | 0.55 (0.21, 1.41) |
|  | Sleep quality | 0.26 (-0.43, 0.96) | 0.17 (-0.25, 0.59) | 2.04 (0.22, 19.31) |
| **Electronic product use (hours/week)** | Total sleep duration | 0.31 (-0.23, 0.85) | 0.24 (-0.21, 0.70) | -9.60 (---, ---) |
|  | Sleep quality | 0.54 (-0.09, 1.18) | 0.36 (0.01, 0.71) | -15.35 (---, ---) |
| **Smoking** | Total sleep duration | -0.29 (-1.08, 0.51) | -0.21 (-0.74, 0.33) | 0.57 (0.20, 1.65) |
|  | Sleep quality | -0.12 (-0.76, 0.52) | -0.10 (-0.65, 0.45) | 0.61 (0.05, 7.25) |
| **Alcohol drinking** | Total sleep duration | -0.19 (-0.87, 0.50) | -0.17 (-0.74, 0.41) | 0.42 (0.04, 4.24) |
|  | Sleep quality | -0.07 (-0.66, 0.52) | -0.07 (-0.63, 0.50) | 0.44 (0.00, 569.12) |
| **Tea drinking** | Total sleep duration | 0.42 (-0.17, 1.02) | 0.29 (-0.13, 0.72) | 17.85 (0.00, ---) |
|  | Sleep quality | 0.74 (0.03, 1.46) | 0.43 (0.12, 0.75) | -27.81 (---, ---) |
| **Coffee drinking** | Total sleep duration | 0.43 (-0.50, 1.36) | 0.31 (-0.30, 0.93) | -6.55 (---, ---) |
|  | Sleep quality | 0.27 (-0.89, 1.43) | 0.19 (-0.50, 0.88) | 2.54 (0.05, 143.90) |
| **Educational level** | Total sleep duration | 0.03 (-0.69, 0.76) | 0.02 (-0.47, 0.51) | 1.07 (0.22, 5.17) |
|  | Sleep quality | 0.51 (-0.09, 1.11) | 0.34 (-0.02, 0.71) | -23.46 (---, ---) |
| **Annual family income (RMB thousand yuan)** | Total sleep duration | 0.36 (-0.15, 0.86) | 0.30 (-0.16, 0.75) | -1.29 (---, ---) |
|  | Sleep quality | 0.30 (-0.32, 0.93) | 0.22 (-0.19, 0.63) | 4.55 (0.01, 2467.33) |
| **Rotating night shift work** | Total sleep duration | 0.28 (-0.55, 1.12) | 0.20 (-0.37, 0.77) | 3.28 (0.00, 2498.94) |
|  | Sleep quality | 1.23 (0.07, 2.40) | 0.61 (0.31, 0.91) | -4.85 (---, ---) |

AP, the attributable proportion due to interaction; CI, confidence interval; RERI, the relative excess risk due to interaction; S, the synergy index.

^a^ Adjusted for age, body mass index, physical activity, electronic product use, abstinence time, smoking, alcohol drinking, tea drinking, coffee drinking, educational level, annual family income, and rotating night shift work, unless a certain covariable is the basis of the interaction.
